# The Song Must Go On: Resilience of the Songbird Vocal Motor Pathway

**DOI:** 10.1371/journal.pone.0038173

**Published:** 2012-06-29

**Authors:** Barish Poole, Jeffrey E. Markowitz, Timothy J. Gardner

**Affiliations:** 1 Department of Biology, Boston University, Boston, Massachusetts, United States of America; 2 Department of Cognitive and Neural Systems, Boston University, Boston, Massachusetts, United States of America; 3 Center of Excellence for Learning in Education, Science and Technology, Boston,Massachusetts, United States of America; Claremont Colleges, United States of America

## Abstract

Stereotyped sequences of neural activity underlie learned vocal behavior in songbirds; principle neurons in the cortical motor nucleus HVC fire in stereotyped sequences with millisecond precision across multiple renditions of a song. The geometry of neural connections underlying these sequences is not known in detail though feed-forward chains are commonly assumed in theoretical models of sequential neural activity. In songbirds, a well-defined cortical-thalamic motor circuit exists but little is known the fine-grain structure of connections within each song nucleus. To examine whether the structure of song is critically dependent on long-range connections within HVC, we bilaterally transected the nucleus along the anterior-posterior axis in normal-hearing and deafened birds. The disruption leads to a slowing of song as well as an increase in acoustic variability. These effects are reversed on a time-scale of days even in deafened birds or in birds that are prevented from singing post-transection. The stereotyped song of zebra finches includes acoustic details that span from milliseconds to seconds–one of the most precise learned behaviors in the animal kingdom. This detailed motor pattern is resilient to disruption of connections at the cortical level, and the details of song variability and duration are maintained by offline homeostasis of the song circuit.

## Introduction

Skilled movement sequences are central to the lives of animals and humans, and the neural control of temporally ordered behaviors is a subject that has attracted intense interest [1]. Hebb theorized that discrete sets of co-active and mutually supporting neurons or “cell assemblies” were the fundamental units of behavior, and that the serial order of action was governed by a sequential chaining of active cell assemblies [2]. The modular structure of phonemes and words as well as the serial order of language naturally lead to the idea that sequential activation of modular cell assemblies could underlie the most sophisticated forms of human cognition.

In the last decade, studies of rodents moving along linear tracks have produced compelling evidence for temporally ordered sequences of neural activity [3], [4]. These sequences reactivate spontaneously in resting and sleeping animals, indicating that temporally ordered sequential state transitions may be intrinsic to the organization of cortico-hippocampal circuits [5]. Experimental identification of chain-like neural activity in behavior can also be found in the learned song of oscine birds, and in the case of the zebra finch, the behavior is highly stereotyped, and the underlying neural patterns precisely timed. The songbird cortical motor nucleus HVC contains inhibitory interneurons, and two classes of excitatory cells that make contacts within HVC and also project to downstream basal ganglia and motor cortex targets [6]. Aligning the spiking of individual projection neurons in HVC to multiple renditions of a song revealed sparse, stereotyped neural firing patterns consistent with the ordered sets of “cell assemblies” predicted by Hebb [7]–[9]. During singing, the excitatory Cells that project from HVC to the pre-motor zone RA (robust nucleus of the arcopallium) fire once per song in a high frequency burst that is time-locked from one song rendition to another [8], [10]. For these cells, timing is preserved throughout the song, with a precision of milliseconds. The sustained propagation of temporally precise, sparsely active cells resembles the patterns produced by a specific model for sequential cell assembly transitions known as the synfire chain [11], [12]. In the simplest version of a synfire chain, each neuron participates in one cell assembly, and each cell assembly is connected anatomically to only one other cell assembly. Cell assemblies are activated, pass the activity to the next cell assembly in the chain, and then shut off.

Due to nonlinearities in the activation function of each cell, the propagation of activity from one cell assembly to the next requires synchronous activation of the neurons in the preceding assembly, and this nonlinearity serves to maintain the temporal precision of the activity front across multiple links in the chain [13]. A stabilizing nonlinearity is also present in the song pre-motor neurons: HVC_RA projection neurons fire an “all-or nothing” burst of high frequency action potentials driven by active calcium processes [11]. In computational models, the strong nonlinearity of the regenerative calcium process can serve to stabilize chain propagation [11].

A second important experiment has confirmed another simple prediction of synfire chain models: for a linear chain of cell assemblies, slowing the time-scale of synaptic transmission should lead to uniform slowing of the sequence of spiking from one cell assembly to the next, thus slowing the song sequence without altering its order or precision. Remarkably, if HVC is cooled in a singing bird, the song slows dramatically without significant changes to other acoustic structure [14].

Little is known about the geometry of connections within HVC. Axon collaterals of single projection neurons ramify within HVC [6], and dye injections in one side of the nucleus densely fills axons on the opposite side, but measures of how the density of collateral synapses falls of with distance have not been performed [15]. The sparse coding and cooling results suggest that principle neurons in HVC could be geometrically organized in chains, which is not an improbable form of anatomical organization–the specific sequence of anatomical connections can arise through spike time dependent plasticity [16]. As a baseline it must be assumed that HVC does not act alone in temporal sequence generation since it forms part of cortico-thalamic and cortico-basal ganglia loops [6], [17]. A chain of neural connections could exist in HVC, or in the cortico-thalamic loop, or a combination of both. Evidence for anatomical chains could in principle be found by detailed anatomical reconstructions [18] or through paired recordings that demonstrate a relationship between spike timing and local connectivity. Neither of these approaches is presently feasible, but simpler experiments can provide constraints on how the circuit functions.

In the present study, we examine the consequences of a large-scale perturbation to the geometry of the song circuit. We demonstrate the following: the song nucleus HVC can be severed in half–generating a complete disconnection of axons that mediate communication between the medial and lateral regions of HVC–without significant long-term disruption of song. Short-term changes to song include an increase in acoustic variability and a slowing of the time-scale of song. However, these changes are rapidly reversed, and the majority of the song recovery process proceeds independently of hearing and singing. This result demonstrates that the circuit geometry underlying song is robust to long-range disconnection, and offline plasticity mechanisms are capable of restoring baseline song in the absence of song behavior.

## Results

**Figure 1:**
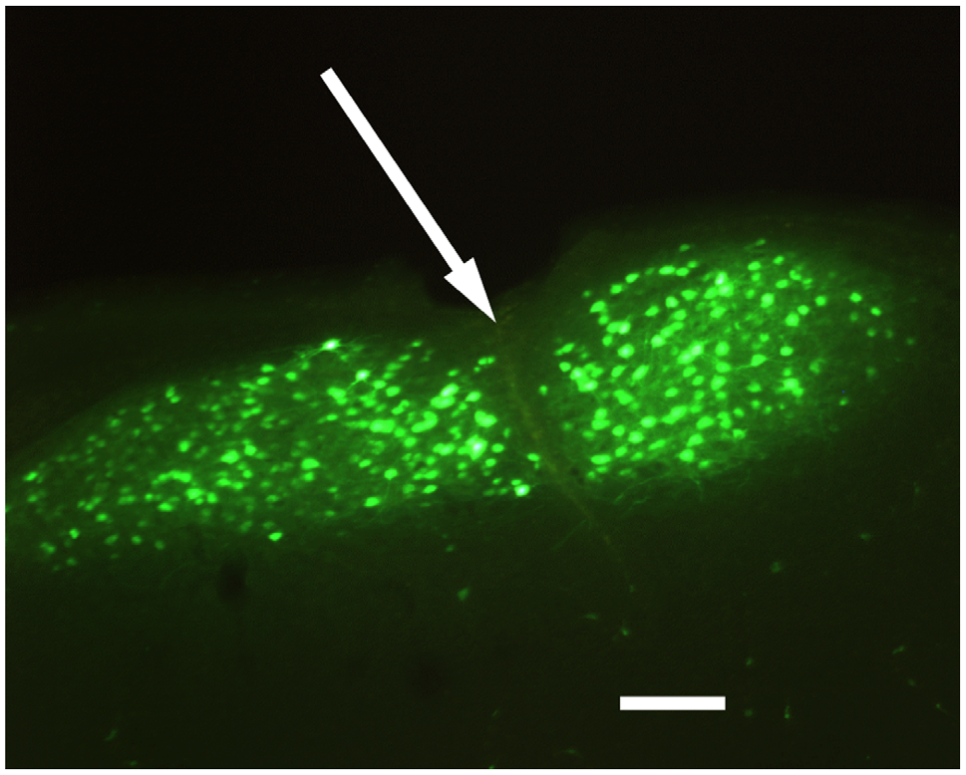
Imaging of the transection site. A 100 micron thick coronal section of HVC in the right hemisphere is shown (lateral to the right, medial to the left). Neurons of HVC are retrogradely labeled from downstream area X with fluorescent dextran. The arrow indicates the location of the transection, fully bisecting the nucleus (scale bar 200 µm).

The song nucleus HVC was completely transected bilaterally in normal adult male zebra finches (N = 5) and transections were confirmed histologically (Fig. 1).

**Figure 2:**
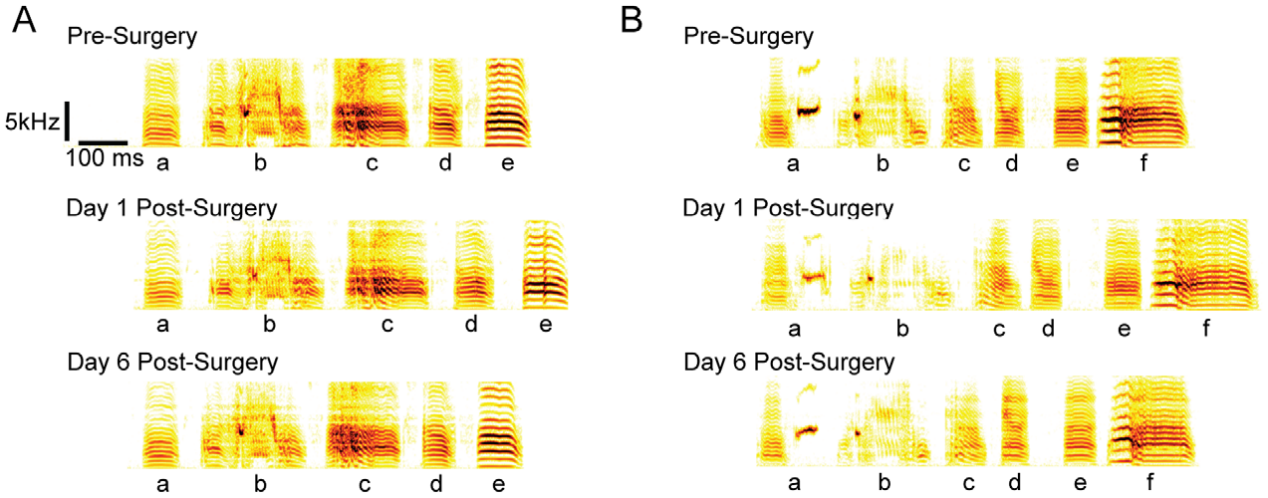
The effects of bilateral HVC transection on song. Representative song motifs from two birds (A and B), with individual syllables labeled. Song is temporally elongated in the first day post-transection. Distortion of spectral structure and an increase in variability is also visible, but both effects disappear by the end of the recovery period.

**Figure 3:**
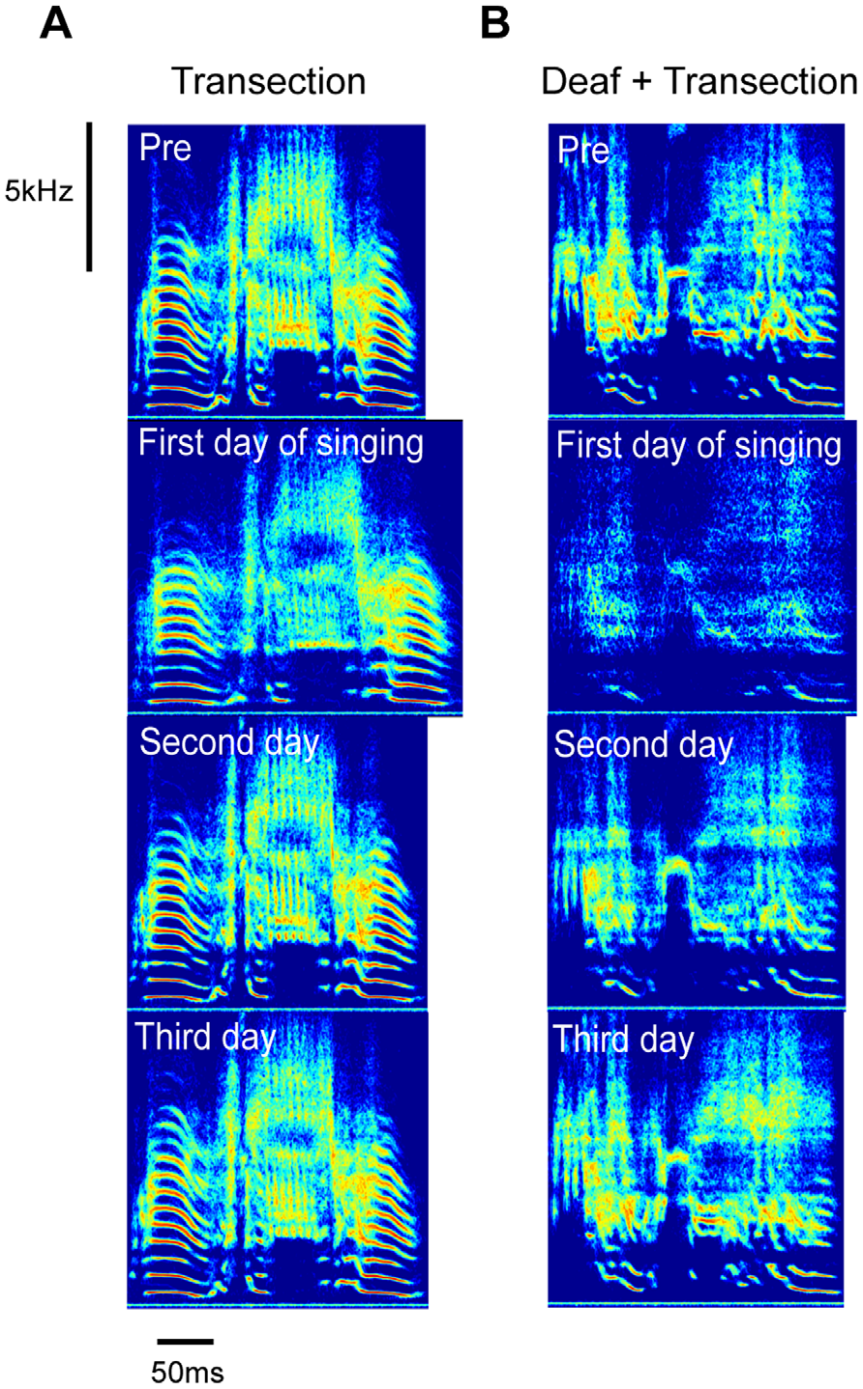
Post-transection changes in individual syllables are corrected within days. (A) Spectral density images (see Materials and Methods) demonstrate the perturbation and recovery of syllable structure following bilateral HVC transection in a male zebra finch. (B) The same effect is observed in a bird deafened before the transection. In both (A) and (B) the progression of syllables is labeled as follows: 1, Pre-surgery; 2, Day 1 of singing post-surgery; 3, Day 2 of singing,; 4, Day 4 of singing.

To first approximation, transection of HVC left song largely intact. This can be seen in sonograms (Fig. 2) as well as spectral density images that superimpose renditions of song in a single time-frequency plot (Fig. 3, see Materials and Methods).

**Figure 4:**
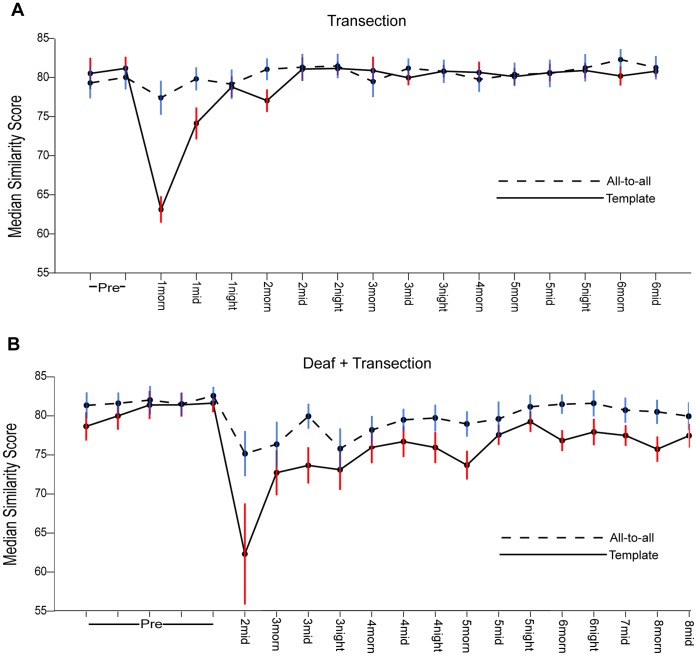
The time-course of syllable recovery. (A) Median similarity scores are plotted over time for both “all-to-all” and “template” analyses of a single syllable following bilateral HVC transection (see Materials and Methods). Error bars in this figure represent the median absolute deviation from the median (MAD), and time points are labeled according to days since transection. (B) Same plot for a bird deafened prior to HVC transection.

**Figure 5:**
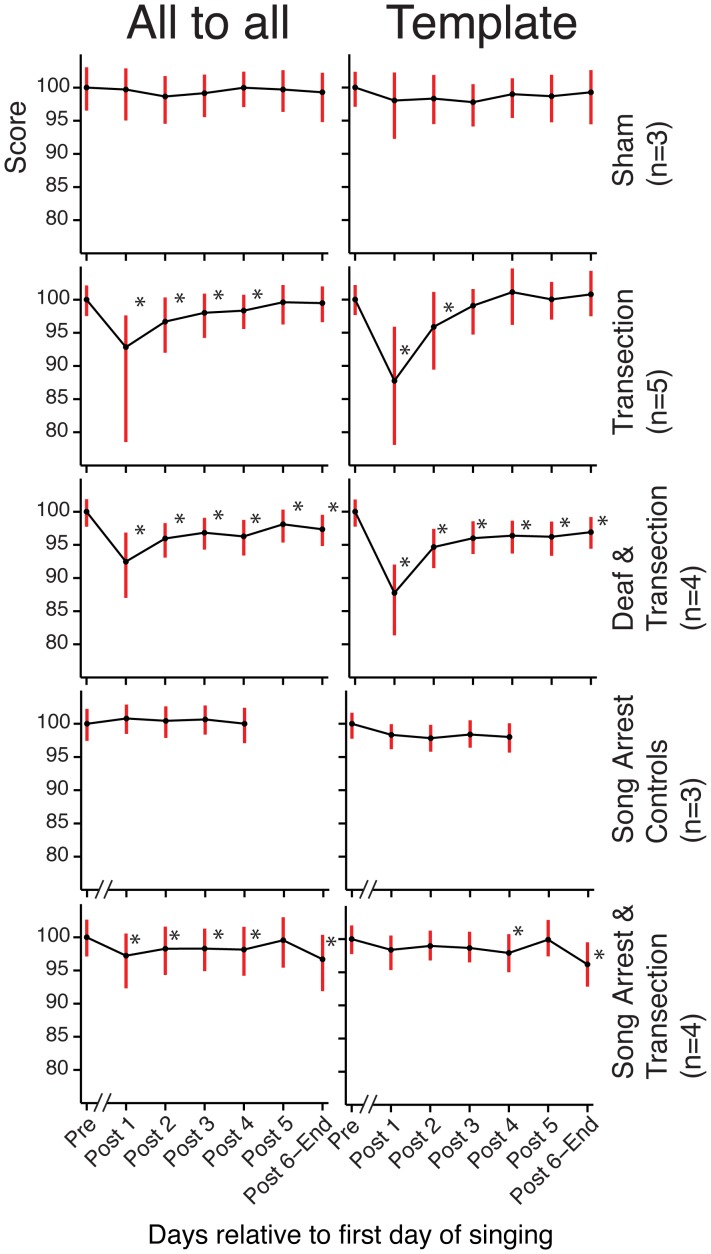
Aggregate grouped plots of similarity scores show the effects of HVC transection in all experimental conditions. (Left) “All-to-all” similarity scores were grouped across birds within each experiment condition into 6 time points: “Pre” is all data collected before the transection, and the time axis is relative to the first day of singing post surgery (either the first or second day post surgery). Median scores were normalized to reflect the percentage of the “Pre” point. (Right) Same plot for the “template” similarity scores. Error bars indicate the first and third quartiles. A break in the abscissa indicates the 4-day gap between the surgery and first post-transection song production for the “Song Arrest” conditions. For all statistical tests in this figure, * indicates p<.05 with Bonferonni correction, one-tailed Monte Carlo permutation test on the difference between medians relative to the sham (10,000 randomizations for each test).

**Figure 6:**
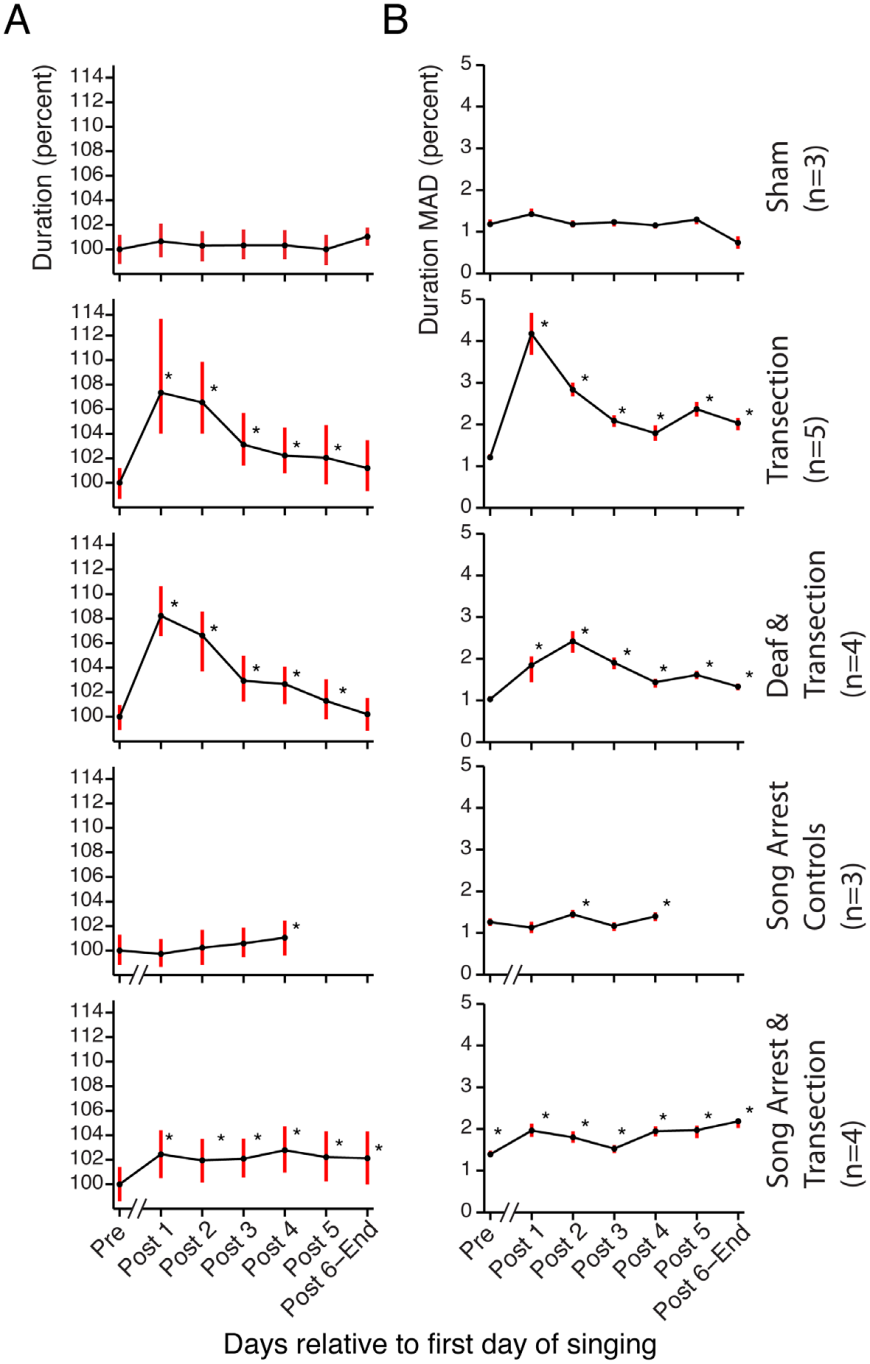
The effect of HVC transection on the duration of a single syllable mirrors the effect on syllable structure. (A) The median duration (given as a percentage of the median “Pre” duration) is plotted for each experimental group. Error bars indicate the first and third quartiles. The statistical tests follow those outlined in the legend for Figure 5. (B) The MAD of the duration is shown for each experimental group. This shows that the variance, in addition to median duration, increases transiently post-transection. Error bars indicate the 95% bootstrap confidence interval of the MAD. Here, * indicates p<.05 with Bonferroni correction, one-tailed Monte Carlo permutation test on the difference between MADs relative to the sham (10,000 randomizations for each test).

Upon closer inspection we found that transection led to a transient increase in song variability. We first quantified this by examining syllable similarities using Sound Analysis Pro [19] focusing on a single syllable type chosen for each bird (see Materials and Methods). Two scores were computed, a “template” score that quantified how spectrally similar each rendition was to a pre-transection template, and an “all-to-all” score that quantifies how stereotyped a group of renditions are. The scores were then grouped by day relative to transection and then by time of day into morning, midday, and evening groups. The top of Figure 4 shows the full time-course of the effect of bilateral HVC transection for a single bird, while Figure 5 (second row) shows summary statistics for the group. The distribution of both the template and all-to-all similarity scores shifts downward relative to the pre-transection baseline distribution immediately post-transection (p<.05, one-tailed permutation test, see legend of Fig. 4) and then recovers within 2–3 days. These Sound Analysis Pro similarity scores must be regarded as a compound measure, impacted by spectral variability, duration, and duration variability. We next examined these components of song stereotypy separately, and found both an increase in spectral variability for many birds (quantified in Fig. S1) and a systematic increase in the time-scale and time-scale variability of song (quantified in Fig. 6A and 6B). The effects on duration, duration variability, and spectral feature variability disappeared within a few days post-transection (Fig. 2, Fig. 6 and Fig. S1).

We next performed transections on deafened birds (N = 4). All birds were adults with a minimum age of 140 days post-hatch in order to avoid the rapid degradation of song that accompanies deafening in young birds [20]. The time course for a single bird is shown in the bottom of Figure 4. Summary statistics reveal, as in the normal-hearing transections, a transient and significant increase in duration (Fig. 6A) and duration variability (Fig. 6B), and also a transient and significant decrease in similarity scores (Fig. 5) post-transection. Over the time-scales we examined, song duration returned to baseline levels in deafened birds, and although template similarity scores recovered dramatically, they did not return completely to baseline.

We next examined whether recovery was dependent on singing. In this experiment birds were prevented from singing for 4 days post-transection (see row 5 in Fig. 5). The structure of the first songs recorded (5 days after surgery) revealed that recovery can proceed subliminally without singing. In particular, songs reached baseline similarity scores in comparison to pre-surgery song templates. A small (relative to the transection groups) but significant shift in the all-to-all scores was detected (Fig. 5.), indicating a slight increase in song variability relative to baseline, as well as a remaining two percent increase in song duration (Fig. 6). By these measures, song recovery proceeded nearly to completion over four days without singing.

To confirm that singing and song recovery were disassociated, we examined whether a correlation between singing rate and recovery existed for the two groups that were allowed to sing during the recovery phase (Normal and Deafened). Song was automatically detected from microphone recordings using custom scripts coupled with visual confirmation (Figure S4, see Materials and Methods). We found no significant correlation between singing rate and recovery rate measured in intervals of days (p>.05).

## Discussion

The principle finding of this study is that the song pattern in zebra finches is robust to complete bilateral transection of the medial and lateral portions of nucleus HVC. The disconnection results in a small-scale transient increase in song variability, duration, and duration variability that is reversed on a time-scale of days. The recovery process for these minor acoustic changes is largely independent of hearing and can proceed subliminally without singing.

Prior studies have reported that song recovery after partial HVC lesions requires intact hearing, with dramatic disorganization of song seen for deafened birds subject to partial HVC lesion. In the present study, the perturbation to song is smaller than that reported for the partial HVC lesions. Due to the effect of an electrolytic lesion on fibers of passage, the size of “microlesions” quantified with Nissl stain can be misleading. For the transections reported here, neurons that lie close to the transection that project to area X can still be backfilled by dye injection after the transection (Fig. 1), indicating that long range connections out of the nucleus can remain intact very close to the transection. The recovery in the absence of hearing and in the absence of singing is not perfect; a small but detectable shift from baseline stereotypy remains in the groups with altered hearing and song prevention. Presumably, the scale of this hearing dependent portion of the recovery would increase with added damage to fibers of passage.

The disruption and recovery of song after transection focuses on a relatively small effect on duration and spectral structure. The central observation is that direct communication between the medial and lateral portions of HVC is not essential for song pattern production. Without additional information about the microstructure of HVC anatomy, we can only speculate why the song pattern is robust to transection. Hahnloser et. al. have shown that nearby HVC_RA projection neurons can fire at distinct times, suggesting that if an anatomical chain in HVC underlies the temporal pattern of song, the chain may not map onto a simple spatial mode such as a travelling wave [8]. Given the density of projection neurons in HVC and the duration of song, 100 projection neurons could code for each 5–10 ms unit of time [21]. If these 100 cells were distributed throughout HVC, then it remains likely that two geometrically intact chains would remain on each side of a bisection. The feedforward redundant geometry of the synfire chain is one network model for temporal sequence production that can, in principle, survive the loss of a large fraction of synapses.

Since HVC and its thalamic input area UVA (uvaeformis) are part of a recurrent cortical-thalamic loop, every stage of the pathway can causally impact activity in all other stages. Cooling of HVC but not the downstream nucleus RA leads to a slowing of song, indicating that biophysical time constants within HVC are fundamental to song timing. It is however possible to separate the serial order of a neural sequence from its timing. The cooling results are consistent with an alternative model in which HVC’s input from the upstream thalamic nucleus UVA is responsible for selecting the specific group of cells that fires at each time point; to be consistent with previous results, the response time of an HVC burst to an UVA kick would need to depend critically on HVC temperature. This alternative model does not propose independent timing circuitry outside of HVC since a delay in HVC response to UVA could propagate through the loop and could slow the next UVA burst at a later time. In this model, the geometry of connections throughout the entire loop could define the specificity of the sequential ordering of song, while leaving critical timing biophysics in HVC. At present, the specificity and frequency of UVA inputs to HVC are not known.

The distinction between the two models described above is a matter of degree. The relative importance of HVC biophysics to song timing, combined with the sparse coding of HVC principle neurons suggests that anatomical chains within HVC are a probable basis for the song sequence. HVC also receives frequent synchronizing inputs from the thalamus that keep the two hemispheres moving in lock-step [22]. A distributed and redundant chain in HVC combined with frequent, specific synchronizing input from the thalamus could create a circuit particularly robust to the transection perturbation described here.

Following HVC transection, song slows, and the recovery of song duration is complete even if the bird is prevented from hearing, and nearly complete even for birds prevented from singing. The slowing of song post-transection is consistent with the view that summation of excitatory input required to trigger action potentials at each step of a chain is delayed due to reduced synaptic drive from the loss of long-range axonal inputs. On the time-scale of recovery that we have observed–3 to 4 days, homeostatic mechanisms could up-regulate the strength of remaining synapses to maintain the equilibrium balance of excitation and inhibition [23]. This would not necessarily require singing since the songbird engages in spontaneous replay of song patterns in sleep [9]; sleep is known to impact the structure of song in juvenile birds [24], and sleep may be involved in offline network and synaptic homeostasis [25]–[27].

The robustness of neural circuits for animal behavior have been appreciated at least since the studies of Lashley, demonstrating a minimal effect of local cortical disconnection that preserved thalamic inputs [28]. The HVC transections prove that even the most stereotyped of animal behaviors can be strikingly resilient to long-range disconnection at the cortical level. If stereotyped serial transitions of cell assemblies underlie aspects of animal behavior and human cognition, then the resilience of neural chain dynamics to noise and other insults is of central importance to the operation of neural memory systems. Pressures to build dynamical patterns that are robust to the vagaries of development or other forms of internal and external noise have reached an extreme in the zebra finch. For this species, mate choice drives song both to increased complexity and increased stereotypy. The resilience of the circuit to perturbation suggests that the bird has resolved these simultaneous constraints by building a high degree of redundancy into the circuit.

## Materials and Methods

Birds were kept on a 14 hr light-dark cycle. Prior to selection birds were housed in sound-proofed recording chambers and allowed to acclimate for at least 4 days, during which time they were screened for singing frequency and distinctiveness of individual syllables. Every bird used in this experiment came from the Boston University breeding colony and was kept in standard conditions (protocol number 11–026). All procedures in this experiment were approved by the Boston University Institutional Animal Care and Use Committee (protocol number 11–027).

### Experimental Groups

Group 1 (n = 5) received a bilateral HVC transection followed 3–7 days later by bilateral injection of retrograde dextran tracer into Area X, directly downstream of HVC. Group 2 (n = 3) acted as a control group, which received sham transections. Group 3 (n = 4) was deafened and allowed to recover for 1–2 weeks before receiving the same bilateral HVC transection and subsequent dye injection. Group 4 (n = 4) underwent transection and was then prevented from singing for 4 days. Group 5 (n = 3) acted as another control group that we prevented from singing in the absence of any transection. Birds’ singing activity was recorded continuously from the time they entered the recording chamber to the time of euthanasia. All subjects were euthanized with urethane and the brains of groups 1, 2, and 4 were extracted and stored in 4% paraformaldehyde solution for at least one day. We then sectioned the brains into 100-micron coronal slices using a Vibratome and mounted them sequentially in glycerol. The full series of sections containing retrogradely labeled HVC cells were inspected by fluorescence microscopy to confirm complete transection (Fig. 1).

### HVC Transections

Sterile HVC transection proceeded by first anesthetizing birds with 1.5–3% isoflurane in oxygen and holding them at a fixed head-angle (approx. 45 degree angle for the upper beak.). Betadine scrub and bupivicaine injection (4 mg/kg) were followed by scalp incision. We made craniotomies over left and right HVC with a dental drill and ophthalmic scalpel, extending the full length of the desired transection. A sharp ophthalmic scalpel (Fine Science Tools) under stereotactic manipulation was moved in the A/P direction at a distance 2.3 mm from the midline, to gradually cut the dura without compressing the brain, then progressively deeper until a transection 1.0 mm deep and 1.0 mm long was complete. We then filled the bilateral craniotomies with Kwik-Cast (World Precision Instruments) silicone elastomer, and sealed the scalp incision with Vetbond (3 M). Meloxicam (1 mg/kg) analgesic was delivered orally for three days post-surgery.

### Dextran Dye Injections

The surgery preparation is identical to that of transection, except we made the craniotomies over Area X bilaterally. A Nanoject II device, with injection needles fashioned out of glass micropipettes, was used to make one to three 23 nL injections of either 488 mm or 555 mm fluorescent dextran dye into Area X. Injections were made either with a single dose in the center of the area, or three or four separate injections around the periphery. The two methods yielded similar patterns of HVC labeling.

### Deafening

Sterile deafening procedure was achieved through bilateral cochlear extraction. Birds were anesthetized with isoflurane (see HVC transections) and an incision in the skin over the external meatus was made and the skin retracted. We then extracted the columella (inner ear bone) with footplate attached with forceps. A fine fire-sharpened tungsten hook was fabricated and then inserted through the oval window and manipulated to grasp and remove the cochlea. Similar to the other procedures, we sealed the skin with Vetbond.

### Control Surgeries

Sham transections were conducted in a manner identical to HVC transections, except that we made the cuts in an area adjacent to HVC at a distance of approximately 3.2 mm from the midline. Cuts made in this way were of the same proportions as those in HVC transections.

### Song Prevention

Prevention of singing was attempted by multiple means for 8 experimental birds–for example, holding the birds on a rotary-platform (Barnstable), which was triggered by a Tucker-Davis RX8 digital signal processor that detected song. We found that automated song prevention did not completely stop the most determined singers, and so we ultimately relied on continual human intervention – we sat next to the cage, and observed the behavior of the birds continuously. When postural cues, introductory notes, or elevated calling rates indicated that a bird was about to sing, the cage was manually shifted or moved to a new location. A maximum of two birds could be effectively monitored at a time, housed in a single cage. In all cases an observer was present for the duration of waking hours during the non-singing week, and during the night automated monitoring of song was used to confirm the absence of singing in the dark.

### Automated Syllable Clustering

Custom MATLAB (Mathworks, Natick, MA) scripts were used for automated syllable clustering. First, a template vocalization of a selected syllable was manually chosen. Then, features from the template and the rest of the data were computed from a ratio of two quantities: the standard sonogram (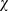 ) of the sound pressure time series 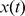, computed with a Gaussian window of time scale 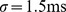, and a sonogram computed with the derivative of the Gaussian window (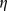) [29].
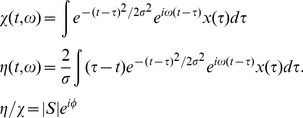
(1)


The complex phase 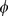 of the ratio 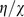 provides an exact measure of the direction of maximal spectral derivative. This direction of maximum spectral derivative has been employed previously in the characterization of zebra finch song, with slightly different mathematical definitions [19]. From these terms, we calculated spectral features: the local power in the sonogram 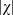, 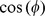 , and a measure of how quickly the local spectral derivative is changing in time 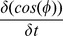 and frequency 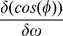. Peaks in the cross-correlation over these features between the template and the data defined potential renditions of the template. As a final step, candidate sounds were plotted in two dimensions manually selected from the set of features defined above, and a decision boundary was drawn by the user, designating a cluster of likely matches to the template. We pre-screened birds with distinct syllable clusters. Quantification was performed for syllable types with clear clustering boundaries at each time point in the behavioral record. Since the acoustic effect of the transection was relatively small, transections did not result in ambiguity in syllable clustering.

### Spectral Density Images

To generate a single image that summarizes the variability of a given vocalization, we computed the superposition of a series of aligned sparse time-frequency representations. Using the ratio defined in (1), a group of sounds were aligned using their peak cross-correlation over the features mentioned above. A specified number of renditions were randomly chosen from the cluster of possible template matches to create an aggregate spectral density image, and a sparse time-frequency representation was calculated for each selected sound [29]. We denote the sparse time-frequency representation,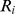, for sound *i*, and then apply the following transformation,
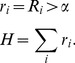
(2)


That is, the magnitude of the sparse time-frequency representation for each sound is thresholded, leaving a series of time-frequency binary images that are subsequently summed across renditions to form a probability density in time and frequency, 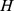.

We applied the above procedure to groups of song data arranged by time of day, based on a 9 AM–11 PM light cycle. 9 AM to 12 PM forms the “morning” group, 12 PM to 4 PM the “midday” group and 4 PM–11 PM the “evening” group.

### Quantification of Syllable Similarity

We used Sound Analysis Pro (version 2.062) [19] in similarity batch mode operating under default parameter settings, which were the following: feature calculation, amplitude baseline 70 dB, frequency range of Wiener entropy and amplitude 430–4300 Hz, dynamic alteration between cepstrum and mean frequency enabled; similarity score options, pitch 1, FM 1, AM 1, entropy 1, goodness 1, time warping tolerance .05. Under these settings, similarity scores were generated to quantify: (1) the variability among renditions of a syllable (generated by all-to all comparisons within a given syllable type), and (2) the likeness of syllable renditions to pre-surgery template renditions throughout a bird’s course of singing post-surgery. These two distinct sets of similarity scores are referred to as “all-to-all” and “template”, respectively.

Both measures were computed for all renditions of a given syllable at each time point. The template comparison in particular can be impacted by changes in either duration or spectral variability, and is included here only to illustrate that all measures of song structure examined show a similar transient perturbation. To demonstrate that some birds undergo an increase in spectral variability independent of song timing, we computed the dispersion of various spectral features that are independent of duration and show their time courses in Figure S1, Figure S2, and Figure-S3.

### Quantification of Syllable Duration

Syllable boundaries were defined by first aligning syllables as described above.

Power threshold crossings in the frequency band 300–1200 Hz defined the onset and endpoint of the syllable precisely. Visual inspection of all labeled boundaries confirmed a low incidence of inaccurate labeling (<5% of syllables).

### Automated Song Detection

To count the number of song bouts for each bird, we conducted an automated survey of the microphone recordings using threshold crossings in the power ratio of the 2000–4000 Hz band versus all frequencies outside that band. Segments of the recording labeled as song bouts were then visually inspected for errors.

## Supporting Information

Figure S1 **Time courses of spectral feature variance for all normal-hearing birds subject to transections–each bird is shown in a separate plot.** All points are labeled relative to the day of the transection. Most birds show a significant increase in spectral variability at the first time point after surgery, followed by a rapid recovery. To compute spectral variability, we first computed the mean entropy, entropy variance, mean amplitude, mean gravity center, mean pitch goodness and mean pitch for all renditions of a given syllable at a given time point (see Materials and Methods for the clustering procedure). These diverse features were then normalized to common units by subtracting the mean of the points preceding the transection and dividing by standard deviations. In the figure we show the median absolute deviation (MAD) of the feature score at each time point, averaged over all features (shown as filled circles). The error bars reflect the 95% bootstrap confidence interval. The p-values were calculated with a one-tailed Monte Carlo permutation test on the difference between MADs between each time point post-transection and all pre-transection time points grouped together (10,000 randomizations per test), * indicates p<.05 with Bonferroni correction.
(TIF)

Figure S2 **Time courses of spectral variance for all transections on deafened birds.** The transection related increase in spectral variability is less consistent in this group of birds. A trend towards increasing variability over time suggests that the acute recovery from transection is superimposed on a deafening related increase in variability. All conventions follow Figure S1.
(TIF)

Figure S3 **Time courses of spectral variance for all shams. All conventions follow Figure S1.**
(TIF)

Figure S4 **Time courses for number of detected songs for each bird (shown by different line colors) in the control and three experimental groups.** Songs were detected through an automated survey (see Materials and Methods), time points are labeled relative to the day of the transection (“Pre” is the median of all points before the transection, and Post 6-End is the median of all points at least 6 days after the transection).
(TIF)
