# Supplementary figures and images for: The Song Must Go On: Resilience of the Songbird Vocal Motor Pathway
Source: PLoS One. 2012 Jun 29;7(6):e38173. doi: 10.1371/journal.pone.0038173 (PMC3387175; doi:10.1371/journal.pone.0038173)

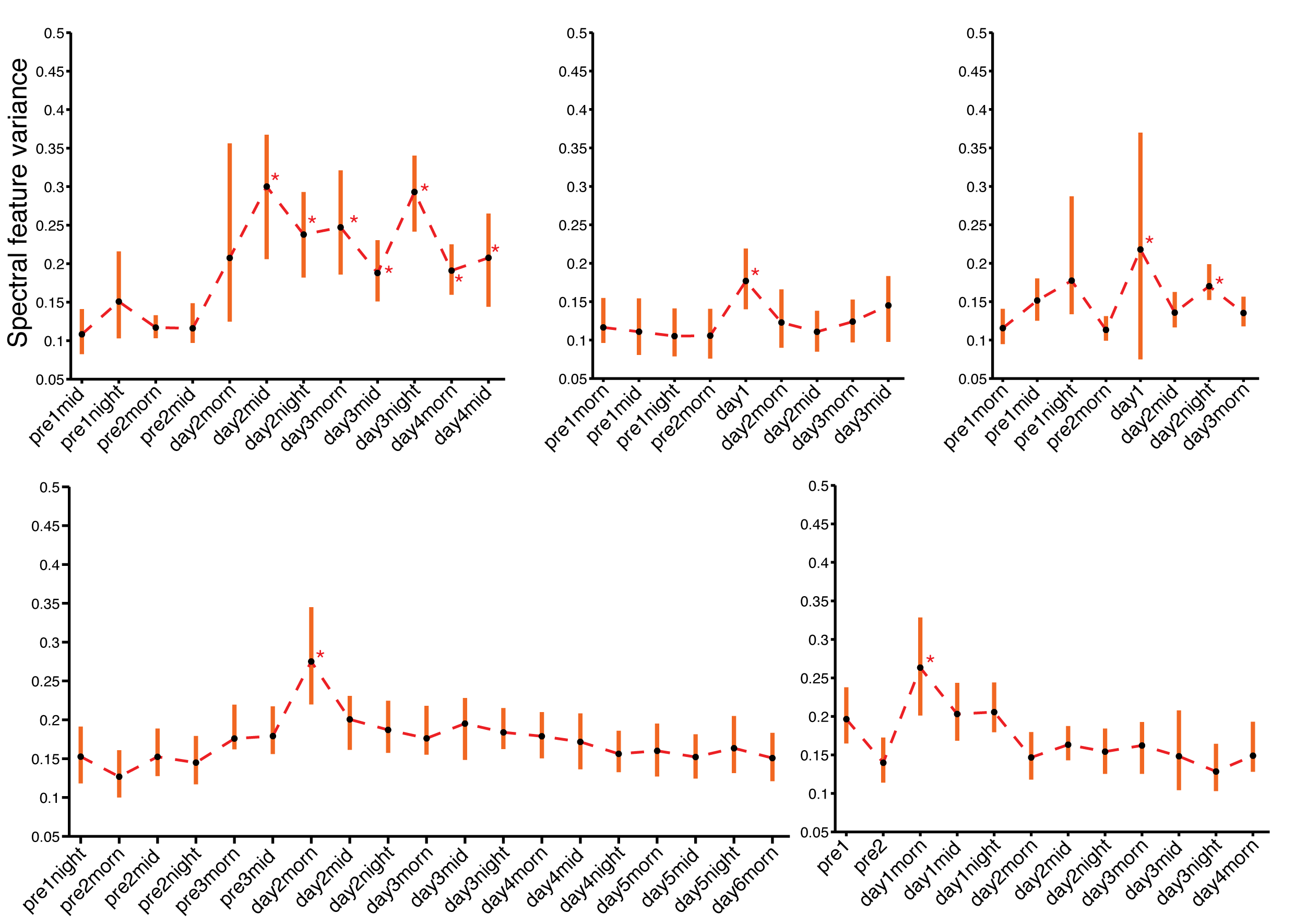

Supplement: Figure S1 — Time courses of spectral feature variance for all normal-hearing birds subject to transections–each bird is shown in a separate plot. All points are labeled relative to the day of the transection. Most birds show a significant increase in spectral variability at the first time point after surgery, followed by a rapid recovery. To compute spectral variability, we first computed the mean entropy, entropy variance, mean amplitude, mean gravity center, mean pitch goodness and mean pitch for all renditions of a given syllable at a given time point (see Materials and Methods for the clustering procedure). These diverse features were then normalized to common units by subtracting the mean of the points preceding the transection and dividing by standard deviations. In the figure we show the median absolute deviation (MAD) of the feature score at each time point, averaged over all features (shown as filled circles). The error bars reflect the 95% bootstrap confidence interval. The p-values were calculated with a one-tailed Monte Carlo permutation test on the difference between MADs between each time point post-transection and all pre-transection time points grouped together (10,000 randomizations per test), * indicates p<.05 with Bonferroni correction. (TIF) [file pone.0038173.s001.tif]

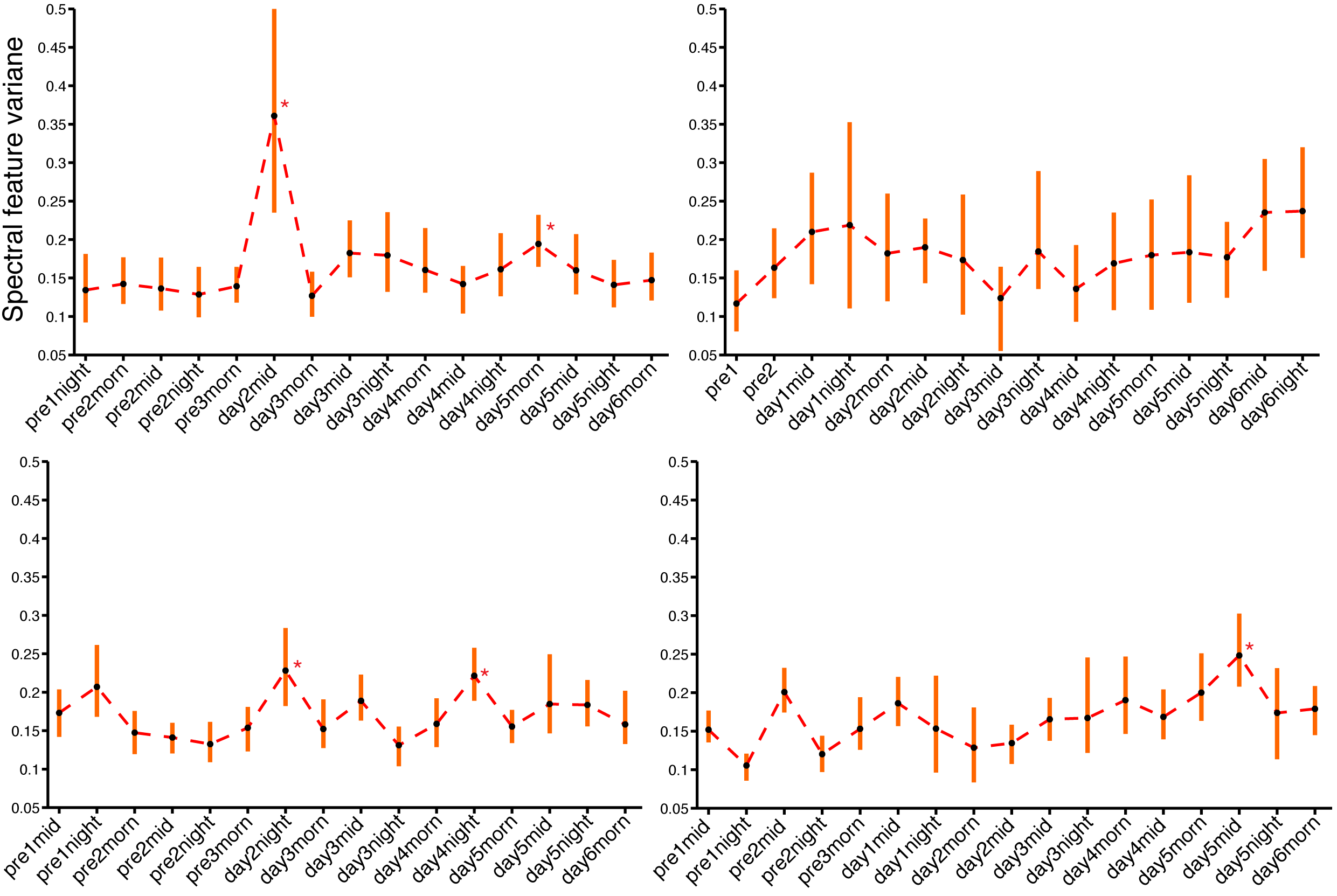

Supplement: Figure S2 — Time courses of spectral variance for all transections on deafened birds. The transection related increase in spectral variability is less consistent in this group of birds. A trend towards increasing variability over time suggests that the acute recovery from transection is superimposed on a deafening related increase in variability. All conventions follow Figure S1. (TIF) [file pone.0038173.s002.tif]

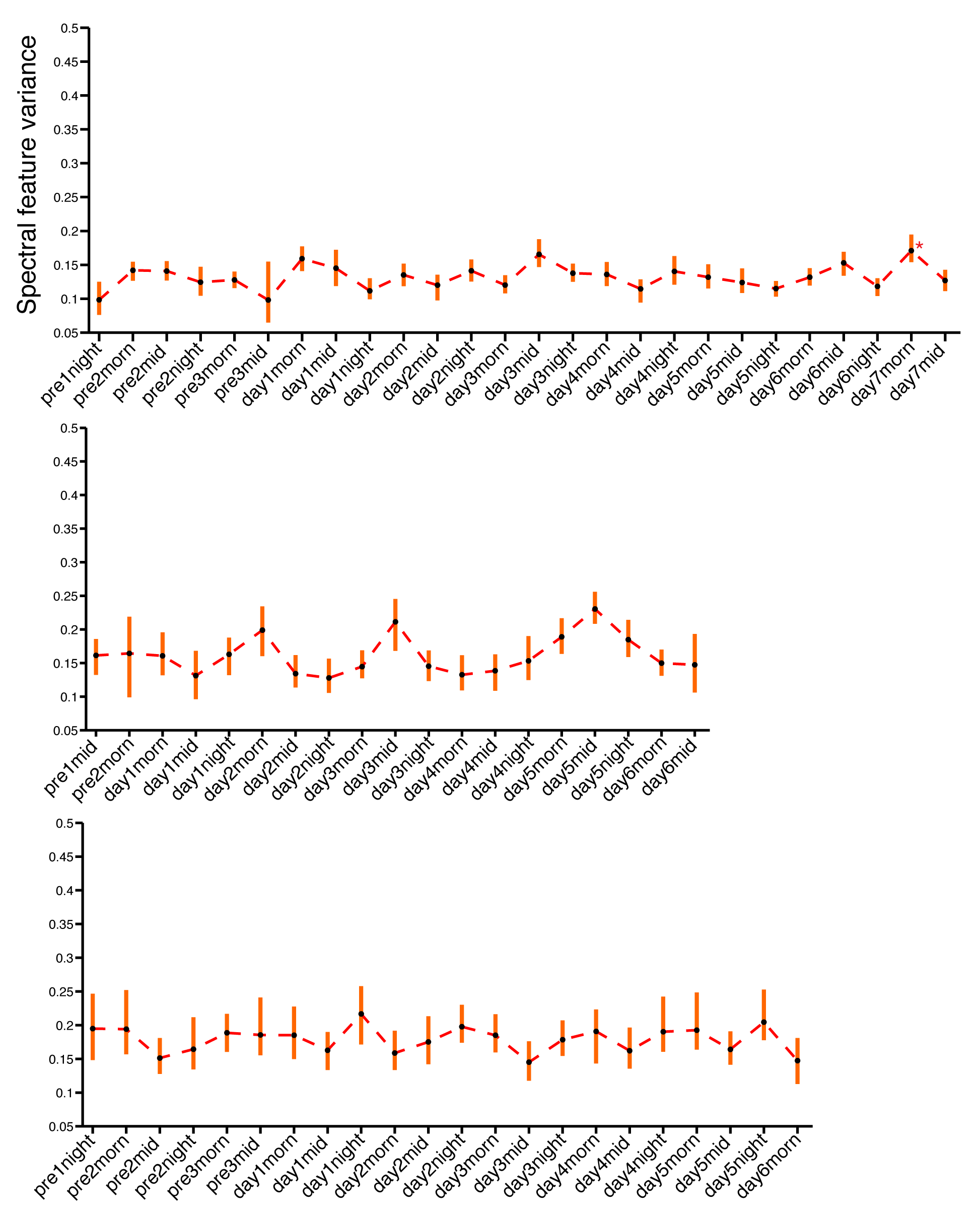

Supplement: Figure S3 — Time courses of spectral variance for all shams. All conventions follow Figure S1. (TIF) [file pone.0038173.s003.tif]

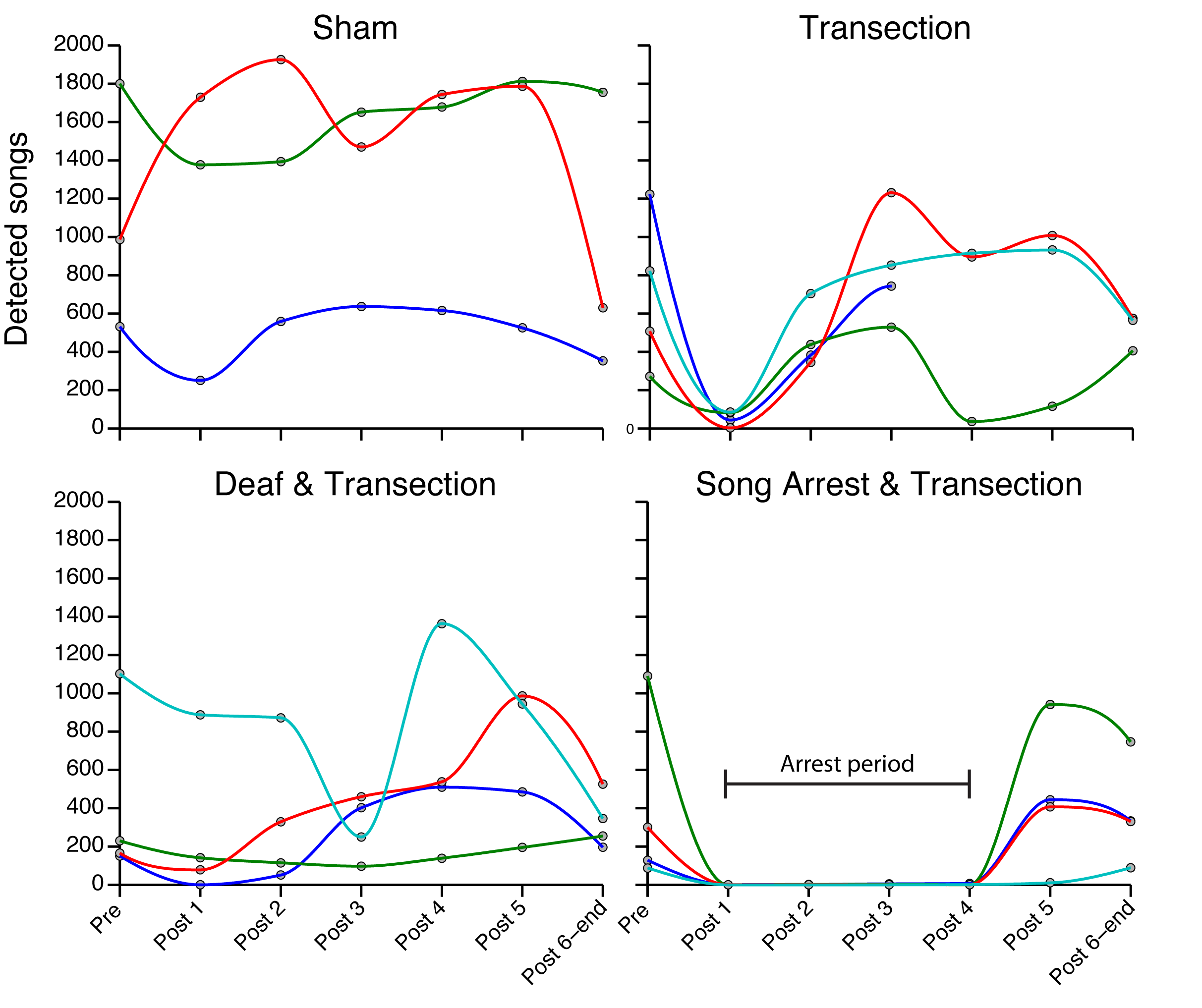

Supplement: Figure S4 — Time courses for number of detected songs for each bird (shown by different line colors) in the control and three experimental groups. Songs were detected through an automated survey (see Materials and Methods), time points are labeled relative to the day of the transection (“Pre” is the median of all points before the transection, and Post 6-End is the median of all points at least 6 days after the transection). (TIF) [file pone.0038173.s004.tif]
